# Thrombotic complications in patients treated with immune checkpoint inhibitors: a retrospective study of incidence, risk factors, and survival outcomes

**DOI:** 10.3389/fimmu.2026.1852772

**Published:** 2026-08-14

**Authors:** Ali Awada, Ali Ghais, Sary Faraj, Ali Tarhini, Joe Rizkallah, Nour Mashmoushi, Souad Susan Sawaf, Nicole Charbel, Jana Haroun, Akel Khaled, Mikel Madi, Katia Kteich, Sara El Mustapha, Mohammad Hassan, Firas Kreidieh

**Affiliations:** 1 Division of Hematology and Oncology, Department of Internal Medicine, American University of Beirut, Beirut, Lebanon; 2 Department of Diagnostic Radiology, American University of Beirut, Beirut, Lebanon; 3 Department of Surgical Oncology, The University of Texas MD Anderson Cancer Center, Houston, TX, United States

**Keywords:** arterial thromboembolism, immune checkpoint inhibitor, real world data, thromboembolism, venous thromboembolism

## Abstract

**Background:**

Immune checkpoint inhibitors (ICIs) have transformed cancer outcomes across multiple malignancies. However, emerging evidence suggests that thrombotic events, including venous thromboembolism (VTE) and arterial thromboembolism (ATE) are serious complications that may negatively affect survival. This study aims to determine their incidence, identify independent risk factors, and evaluate effects on outcomes and survival.

**Methods:**

We retrospectively reviewed adult patients treated with ICIs at a tertiary academic center. Thrombotic events were classified as VTE or ATE occurring from ICI initiation through follow-up. Clinical, laboratory, and treatment-related variables were collected. Kaplan–Meier methods and multivariable Cox proportional hazards models were used to evaluate survival impact and identify independent predictors.

**Results:**

Among 750 patients treated with ICIs, 125 (16.6%) thrombotic events occurred over a median follow-up of 25 months. Of these events, 105 (84.0%) were VTE and 20 (16.0%) were ATE. Of all thrombotic events, 38% occurred within the first 6 months, 56% within 12 months, 67% within 18 months, and 74% within 24 months. Independent predictors of TE included prior VTE (HR 1.84, 95% CI 1.12–3.01; p = 0.015), lower baseline hemoglobin (HR 0.88, 95% CI 0.80–0.99; p = 0.036), and older age at immunotherapy initiation (HR 1.07 per year increase, 95% CI 1.04–1.11; p < 0.001).Thrombotic events were associated with significantly worse overall survival (OS) (log-rank p < 0.001) and remained independently associated with mortality in multivariable analysis (HR 2.55, 95% CI 1.89–3.43; p < 0.001). Additionally, prior thrombotic events (HR 1.86, 95% CI 1.29–2.69; p = 0.001) and a Khorana score ≥2 (HR 1.31, 95% CI 1.13-1.50, p<0.001) were independently associated with worse OS.

**Conclusions:**

Thrombotic events are common in patients receiving ICIs and are independently associated with worse survival. Risk-based and personalized assessment of thrombotic events can help guide monitoring and management strategies.

## Highlights

- Thrombotic events are increasingly recognized with immune checkpoint inhibitors (ICIs). While clinical trials report low rates, real-world data suggest a substantially higher incidence, with limited understanding of timing, risk factors, and survival impact.
- In 750 patients, 16.6% developed thrombosis, with early venous and later arterial events. Prior thromboembolism, older age at treatment initiation, and low hemoglobin predicted risk. Thrombosis strongly worsened survival, especially with Khorana score ≥2.
- This study highlights need for systematic risk assessment, monitoring, and personalized prevention strategies in ICI-treated patients.

## Introduction

The advent of immune checkpoint inhibitors (ICIs) has revolutionized modern oncology by harnessing the host immune system to achieve lasting antitumor responses across a wide variety of cancers. By targeting inhibitory pathways such as programmed cell death protein-1 (PD-1), programmed death-ligand 1 (PD-L1), and cytotoxic T-lymphocyte–associated antigen-4 (CTLA-4), ICIs restore T-cell activation and enhance immune-driven tumor control (1, 2). Despite their clinical efficacy, treatment outcomes vary greatly. Efficacy depends on tumor-intrinsic factors (such as mutational burden and PD-L1), host factors (immune microenvironment, microbiome, comorbidities), and treatment variables (combination strategies, timing) (3, 4). Furthermore, immune-related adverse events may compromise both treatment continuation and survival outcomes (5–7). Better understanding of these determinants is crucial for optimizing patient selection, minimizing toxicity, and improving personalized ICI-based cancer therapy.

Cancer-associated thrombosis is driven by multiple overlapping mechanisms that fulfill all three components of Virchow’s triad: hypercoagulability, endothelial injury, and venous stasis. Emerging evidence suggests that ICIs may increase this risk of thrombosis through endothelial activation, systemic inflammation, autoimmunity, vasculitis, and T cell imbalance. To date, only a few studies have reported this association. Khorana et al. reported higher clot rates in patients with cancer on ICIs, especially those with advanced non-small cell lung cancer, with unique risk factors (6). Additionally, retrospective analyses have linked ICIs to arterial thrombotic events (ATE), suggesting immune-mediated vascular injury and inflammation as potential mechanisms (8). Recent reviews and pharmacovigilance studies further reinforce this association, highlighting immune activation, endothelial dysfunction, and inflammasome signaling as plausible pathways (9). Collectively, these findings underscore the need for heightened awareness and tailored thrombosis risk assessment in the era of cancer immunotherapy.

The timing and features of TE varies across ICI-induced thrombosis. Cohort studies show venous thromboembolism (VTE) is more common within 2–3 months of starting ICI therapy. Large studies in lung and other cancers find VTE incidences of 10–15%, with the highest risk soon after treatment begins, reflecting cancer hypercoagulability and immune activation (10, 11). ATE like myocardial infarction and stroke occur less often with variable timing. Retrospective data link these events to traditional cardiovascular risk factors and suggest they can develop later during ICI therapy or in patients with pre-existing vascular disease (10, 12).

Despite increasing recognition of ICI–associated thrombosis, there remains a significant gap in the literature. Randomized clinical trials report low cumulative thrombosis rates of approximately 3–4%, with VTE at 2.7% and ATE at 1.1%, likely underestimating true risk (13). These trials focused on specific patients and major adverse events, often missing nonfatal or delayed events. Real-world studies show higher thrombotic rates, highlighting a gap with trial data. In this retrospective study, we aim to determine the incidence, timing, predictors, and impact of thrombotic events on survival and treatment outcomes in order to improve risk management.

## Methods

### Design and study population

This is a single-center retrospective cohort study at the American University of Beirut Medical Center (AUBMC). Patients included in the study were adults with solid tumors who received at least one dose of an immune checkpoint inhibitor (ICI) between January 1, 2018, and July 31, 2025. Eligible ICI agents were pembrolizumab, nivolumab, atezolizumab, avelumab, ipilimumab, and durvalumab, administered as monotherapy or in combination, and categorized by drug class and regimen. The index date was defined as the date of first ICI administration. Patients were followed from ICI initiation until last documented follow-up.

### Data collection and covariates

Clinical, laboratory, and treatment-related variables were extracted from the electronic medical record. Baseline variables collected at ICI initiation included age, sex, body mass index (BMI), comorbidities (including hypertension, diabetes mellitus, dyslipidemia, coronary artery disease, chronic kidney disease),Charlson Comorbidity Index (CCI), Eastern Cooperative Oncology Group (ECOG) performance status, smoking status, tumor type, disease stage, prior or concurrent treatment history (systemic chemotherapy, radiotherapy, targeted therapy), history of thrombotic events, and baseline use of antithrombotic therapy (anticoagulants and/or antiplatelet agents, recorded by medication class). ICI exposure variables included regimen and class, start and end dates, and number of cycles administered.

Baseline thrombotic risk was obtained using the Khorana score, calculated from its component variables (including platelet count, hemoglobin level, leukocyte count, and use of hormone therapy, as applicable) collected at ICI start date, along with other documented thrombosis risk factors (including hormonal therapy, erythropoietin hormone therapy, and chronic inflammatory/autoimmune diseases). The Khorana score was used as a baseline thrombotic risk comparator rather than as a predictive model and was evaluated in an exploratory manner.

### Outcomes and definitions

The primary endpoint was incidence of thrombotic events (TE) following ICI initiation, assessed across consecutive 6-month intervals (0–6, 6–12, 12–18, and 18–24 months) from the index date through follow-up. TEs were classified as VTE or ATE. VTE included deep vein thrombosis (DVT) and pulmonary embolism (PE). ATE included myocardial infarction (MI), ischemic stroke, and peripheral arterial thrombosis/occlusion. For events that occurred, we recorded the date of the first TE, TE category (VTE vs ATE), ATE subtype (MI, stroke, peripheral arterial), and whether recurrent TE occurred during follow-up. Thrombotic events were identified and confirmed through review of radiology reports documenting objective imaging findings. Clinically suspected events without imaging confirmation were excluded.

The secondary outcomes included overall survival (OS). OS was defined as the time from ICI initiation to death from any cause, with patients censored at the date of last follow-up if neither event occurred.

### Statistical analysis

Baseline characteristics were summarized using descriptive statistics. The incidence of TE was calculated within each predefined 6-month interval among patients at risk at the start of each interval and reported overall and stratified by TE type (VTE vs ATE). Time-to-event analyses were performed to evaluate factors associated with development of a first TE after ICI initiation. Univariable Cox proportional hazards models were used to screen candidate predictors, followed by multivariable Cox regression to identify independent predictors while adjusting for clinically relevant covariates. Covariates were selected *a priori* based on clinical relevance and prior literature; univariable analyses were used to explore associations and inform multivariable model construction.

Kaplan–Meier methods were used to estimate OS and to compare OS across groups as appropriate, with corresponding multivariable Cox models used to evaluate associations between TE (and TE subtype) and OS while adjusting for confounders. To account for immortal time bias, thrombotic events were modeled as time-dependent covariates in Cox proportional hazards models evaluating OS; this time-dependent Cox HR is the primary inferential estimate for the TE–OS association. To provide a descriptive visual comparison while mitigating immortal time bias, landmark analyses at 6 and 12 months from ICI initiation were performed. Median follow-up was estimated by the reverse Kaplan–Meier method.

All analyses were performed using IBM SPSS statistics (IBM Corp., Armonk, NY, USA). Missing data were infrequent and were handled using complete-case analysis. Statistical tests were two-sided, and p values <0.05 were considered statistically significant.

### Ethics approval

This study was approved by Institutional Review Board of the American University of Beirut (IRB approval number: BIO-2024-0295). The requirement for informed consent was waived due to the retrospective nature of the study. The study was conducted in accordance with the principles of the Declaration of Helsinki.

## Results

### Patients’ characteristics

A total of 750 patients were included in the study. Baseline demographic and clinical characteristics are summarized in **Table 1**. The median age at diagnosis was 70 years [interquartile range (IQR) 62-77], and the median body mass index (BMI) was 25.8 kg/m^2^ (IQR, 23-29). Overall, 485 (64.7%) were males. Most patients had stage IV disease (71.3%), with non-small cell lung cancer being the most common diagnosis (44.1%). The majority of patients in our cohort had a preserved functional status. An Eastern Cooperative Oncology Group (ECOG) performance status of 0 was recorded in 345 patients (67.0%), while 141 patients (27.4%) had an ECOG of 1. Regarding comorbidity burden, 124 patients (16.5%) had a Charlson Comorbidity Index (CCI) of 0-5, while 621 patients (82.8%) had a CCI score>6. A history of autoimmune disease was documented in 50 patients (6.7%), and 575 patients (76.6%) were classified as current or former smokers. Prior thromboembolic events were reported in 75 patients (10.0%) before treatment initiation. In terms of medication use, 96 patients (12.8%) were on chronic anticoagulation therapy, 188 (25.0%) were on antiplatelet therapy, and 15 patients (2.0%) were on hormonal therapy. Concerning treatment regimens, the most commonly administered immunotherapy was pembrolizumab (55.2%), followed by nivolumab (17.4%). Combination immunotherapy was administered to 79 (10.6%) of patients in this cohort. Data regarding systemic therapy showed that 473 patients (63.0%) received concurrent chemotherapy, 360 (48.1%) received concurrent radiotherapy, and 76 (10.2%) received concurrent targeted therapy. Surgical resections of primary tumors were performed in 278 patients (37.1%).

**Table 1:** Baseline demographic and clinical characteristics (n=750).

Demographics	
BMI, median (IQR)	25.8 (23-29)
Age at initiation of immunotherapy, median (IQR)	66 (59-74)

Characteristic	n (%)
Gender	
Male	485 (64.7)
Female	265 (35.3)
History of autoimmune disease	
Yes	50 (6.7)
No	700 (93.3)
Diagnosis	
NSCLC	331 (44.1)
Gastrointestinal cancers	67 (8.9)
Bladder Cancer	63 (8.4)
Renal Cell carcinoma	53 (7.1)
Melanoma	51 (6.8)
Hepatobiliary cancer	51 (6.8)
SCLC	36 (4.8)
Breast Cancer	26 (3.5)
Head and Neck Cancer	19 (2.5)
Gynecological	17 (2.3)
Genitourinary	8 (1.1)
Others	28 (3.7)
Stage IV disease	
Yes	535 (71.3)
No	215 (28.7)
ECOG performance status	
0	345 (46.0)
1	141 (18.8)
2	21 (2.8)
3	7 (0.9)
4	1 (0.1)
Missing	235 (31.3)
Charlson comorbidity index	
0-5	124 (16.5)
>6	621(82.8)
Missing	5 (0.7)
Khorana score at treatment initiation	
0-1	680 (90.6)
≥2	70 (9.4)
History of smoking	
Yes	575 (76.6)
No	175 (23.4)
History of prior TE	
Yes	75 (10.0)
No	675 (90.0)
On anticoagulation therapy	
Yes	96 (12.8)
No	654 (87.2)
Type of anticoagulation therapy	
Factor X inhibitors	47 (6.2)
Low Molecular Weight Heparin	21 (2.8)
Warfarin	4 (0.5)
Direct Thrombin Inhibitors	4 (0.5)
Unfractionated Heparin	2 (0.25)
Fondaparinux	1 (0.1)
Not Recorded	17 (2.2)
On antiplatelet therapy	
Yes	188 (25.0)
No	562 (75.0)
Type of antiplatelet therapy	
Aspirin	115 (15.4)
P2Y12 Inhibitors	40 (5.3)
Combination Therapy	17 (2.2)
Not Recorded	16 (2.1)
On hormonal therapy	
Yes	15 (2.0)
No	735 (98.0)
Prior surgery for primary cancer	
Yes	278 (37.1)
No	472 (62.9)
Type of immunotherapy	
Pembrolizumab	414 (55.2)
Nivolumab	131 (17.4)
Atezolizumab	76 (10.1)
Durvalumab	46 (6.2)
Ipilimumab	4 (0.5)
Combination	79 (10.6)
Concurrent chemotherapy	
Yes	473 (63.0)
No	277 (37.0)
Concurrent radiotherapy	
Yes	360 (48.1)
No	390 (51.9)
Concurrent targeted therapy	
Yes	76 (10.2)
No	674 (89.8)

BMI, body mass index; NSCLC, non-small cell lung cancer; SCLC, small cell lung cancer; ECOG, Eastern Cooperative Oncology Group.

### Incidence of thromboembolic events

With a median follow-up duration of 25 months, TE occurring after treatment initiation were observed in 125 patients (16.6%). Among these events, VTE constituted the majority. Overall, a total of 105 patients experienced venous events; 63 patients had DVT on Doppler ultrasound, 24 patients had a PE by computed tomography pulmonary angiography, and 18 patients experienced both. ATE were less frequent, documented among 20 patients (2.66%). Stroke was the most commonly reported arterial event, affecting 13 patients, followed by MI in 7 patients. Results are summarized in **Table 2**.

**Table 2:** Incidence of thromboembolic events (TE) after immunotherapy initiation.

Total number of TE n (%)	125 (16.6)		
Venous Thromboembolism (VTE)	105 (14.0)	Deep vein thrombosis (DVT)	63 (8.4)
		Pulmonary Embolism (PE)	24 (3.2)
		DVT + PE	18 (2.4)
Arterial Thromboembolism (ATE)	20 (2.6)	Stroke	13 (1.7)
		Myocardial Infarction	7 (0.9)

**Figure 1:**
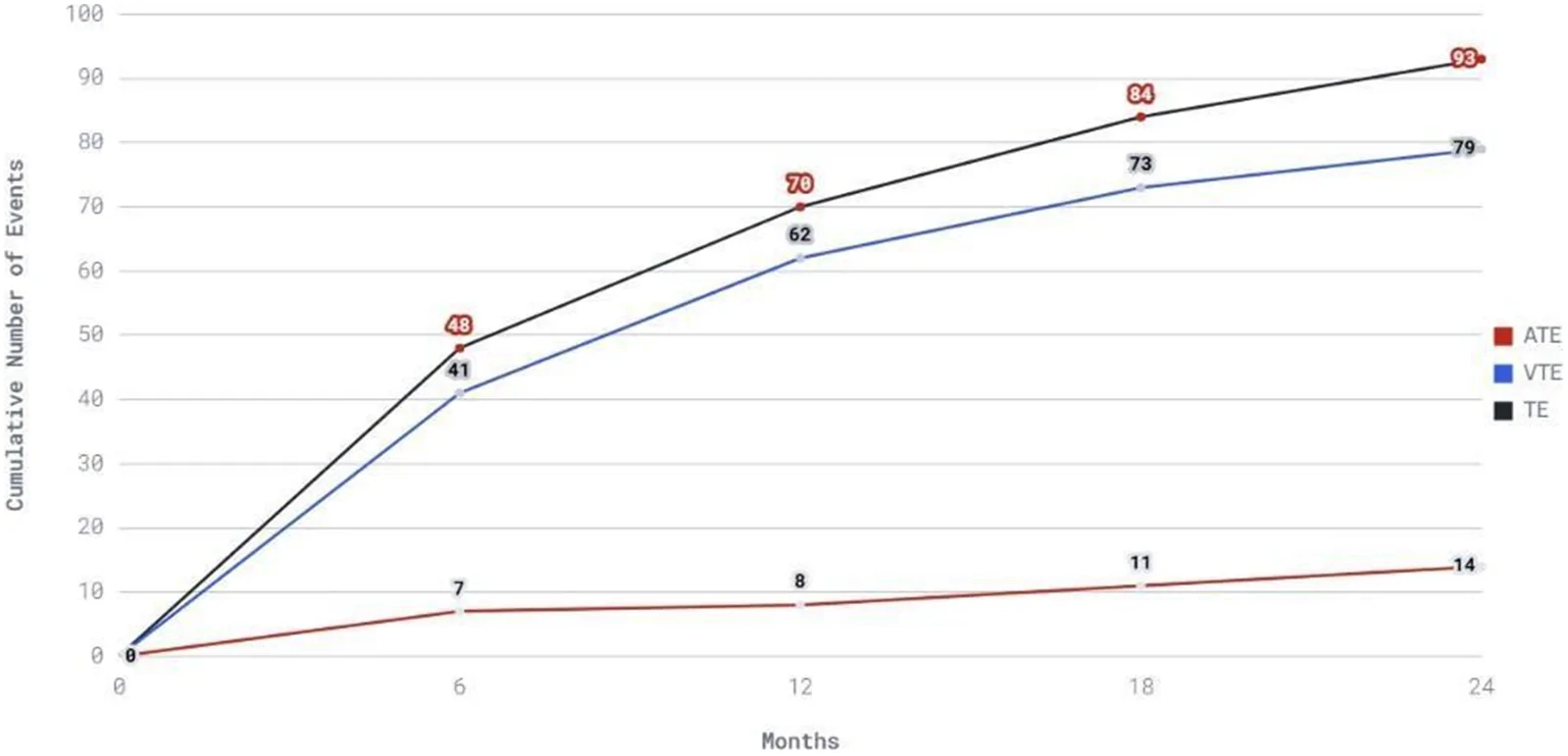
Incidence of TE, VTE, and ATE throughout the follow-up period.

In our cohort, the cumulative incidence of TE was 48 events (38% of total events) at 6 months, 70 at 12 months (56%), 84 at 18 months (67%), and 93 at 24 months (74%) (**Figure 1**).

### TE risk factors in patients receiving ICIs

Univariate analyses were performed to identify baseline patient characteristics and treatment-related factors associated with the risk of TE occurrence following initiation of ICIs. Variables associated with TE risk included older age at immunotherapy initiation (HR 1.028, 95% CI 1.01-1.04, p<0.001), history of any thromboembolic event (HR 2.15, 95% CI 1.33-3.48, p=0.002), history of atrial fibrillation (HR 2.02, 95% CI 1.24-3.31, p=0.005), and a lower baseline hemoglobin (HR 0.86, 95% CI 0.78-0.95, p=0.005). Other variables including patients and disease characteristics showed no significant association (**Supplementary Table 1**).

After adjusting for confounders, multivariable analysis identified several independent predictors of TE occurrence. These included a history of any thromboembolic event (HR 1.84, 95% CI 1.12-3.01, p = 0.015), lower baseline hemoglobin (HR 0.88, 95% CI 0.80-0.99, p = 0.036), and older age at immunotherapy initiation (HR 1.07, 95% CI 1.04-1.11, p < 0.001). The results of the multivariable Cox regression analysis are summarized in **Table 3**.

**Table 3:** Univariate and multivariate Cox regression analysis of factors associated with the occurrence of thromboembolic events.

Variable	Univariate		Multivariate	
	HR (95% CI)	p-value	HR (95% CI)	p-value
History of any prior TE event	2.15 (1.33-3.48)	0.002	1.84 (1.12-3.01)	0.015
age at immunotherapy initiation	1.028 (1.01-1.04)	0.001	1.07 (1.04-1.11)	<0.001
History of atrial fibrillation	2.02 (1.24-3.31)	0.005	–	–
Baseline hemoglobin	0.86 (0.78- 0.95)	0.005	0.88 (0.80-0.99)	0.036

### TE and association with survival outcomes

**Figure 2:**
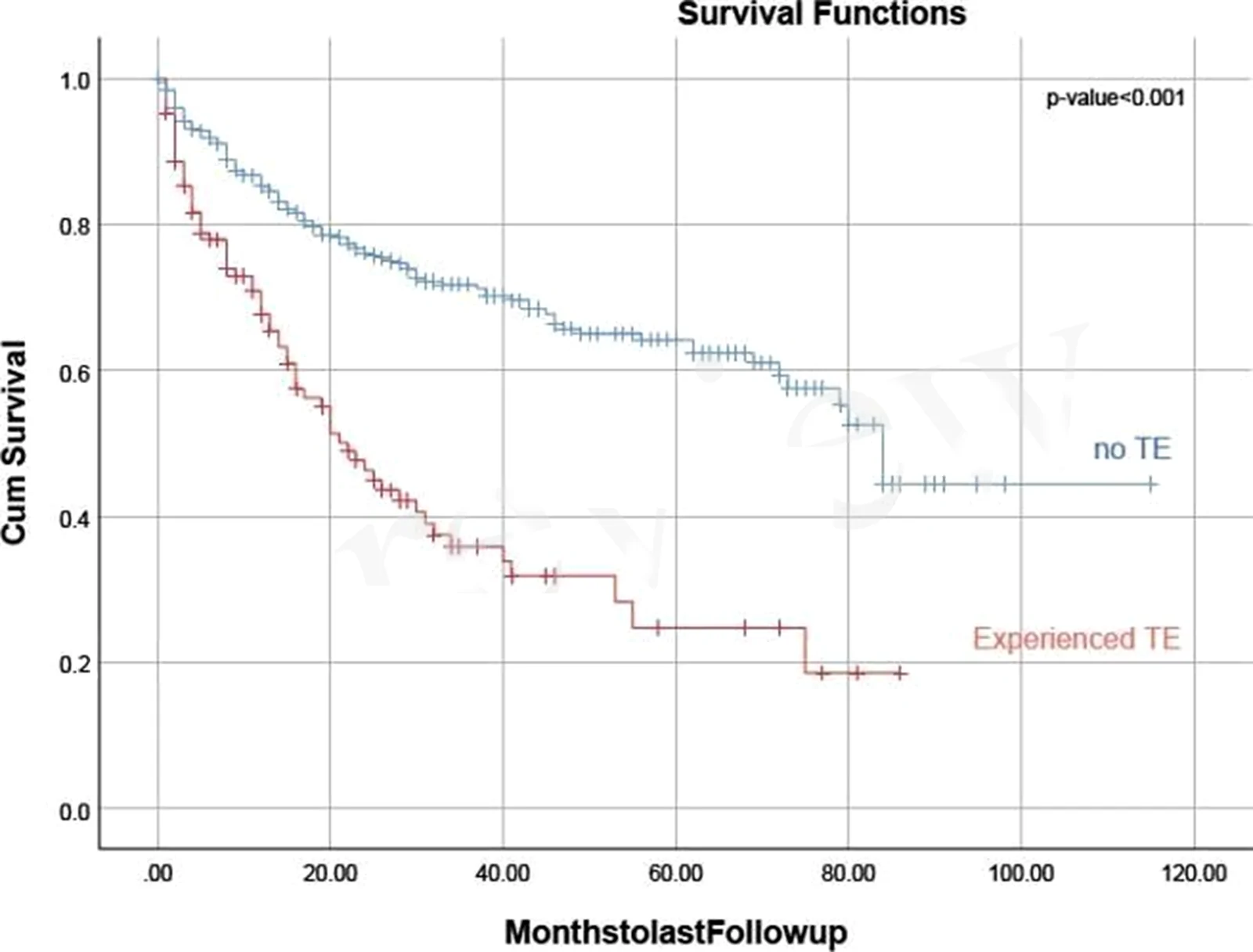
Kaplan–Meier survival curves according to thromboembolic event (TE).

Kaplan-Meier analysis demonstrated significantly reduced survival among patients who developed TE after treatment initiation (log-rank p<0.001). This Kaplan–Meier comparison uses TE status as a fixed baseline split and is presented as a descriptive illustration only; the time-dependent Cox model results (below) are the primary inferential estimates. The median OS for patients with TE was 21.0 months (95% CI 16–31) compared with 84 months (95% CI 73–∞) for those who did not experience TE (**Figure 2**). The 95% CI upper bound of infinity for the no-TE group confirms this estimate is tail-driven: only 73, 36, and 13 patients remained at risk at 60, 72, and 84 months, respectively (**Supplementary Table 3**). The no-TE group was 67.3% Stage IV and a 39.3% rate of complete remission or no evidence of disease at last follow up was observed. Reverse Kaplan–Meier–estimated median follow-up for the no-TE group was 24.0 months (95% CI 22–27; IQR 7–33 months), and for the TE group was TE group 32.0 months (95% CI 25–41; IQR 4–26).

The occurrence of TE remained significantly associated with worse OS in multivariable analysis using a conventional fixed-covariate Cox model. To account for immortal time bias, the primary analysis modeled TE as a time-dependent covariate; this yielded HR 2.41 (95% CI 1.76–3.29; p < 0.001), which is reported in **Table 4** and should be considered the primary inferential estimate. Clinical variables associated with worse survival in univariate analysis included history of any prior TE event (HR 2.23, 95% CI 1.55-3.19, p<0.001), occurrence of TE after treatment initiation (HR 2.69, 95% CI 2.02-3.59, p <0.001), history of hypertension (HR 1.36, 95% CI 1.03-1.78, p=0.02), history of atrial fibrillation (HR 1.682, 95% CI 1.12-2.50, p=0.01), a lower hemoglobin level (HR 0.83, 95% CI 0.77-0.89, p<0.001),Khorana score≥2 (HR 1.70, 95% CI 1.11-2.67, p=0.014) and cancer stage (HR 1.84, 95% CI 1.38-2.45, p<0.001). Other variables including patients and disease characteristics showed no significant association (**Supplementary Table 2**).

**Table 4:** Univariate and multivariate Cox regression analysis of factors associated with overall survival.

Variable	Univariate		Multivariate	
	HR (95% CI)	p-value	HR (95% CI)	p-value
History of any prior TE event	2.23 (1.55-3.19)	0.04	1.55 (1.02-2.35)	p=0.04
Occurrence of TE after treatment initiation	2.69 (2.02-3.59)	<0.001	2.41 (1.76-3.29)	<0.001
History of hypertension	1.36 (1.03-1.78)	0.02	1.23 (0.92-1.64)	0.14
History of atrial fibrillation	1.682 (1.12-2.50)	0.01	1.27 (0.80-2.01)	0.30
Khorana score≥2	1.70 (1.11-2.67)	0.01	1.25 (1.07-1.46)	0.004
Cancer Stage	1.84 (1.38-2.45)	<0.01	1.68 (1.25-2.26)	<0.001

After adjusting for potential confounders, variables that remained significantly associated with survival in multivariable analysis included a prior history of any TE event (HR 1.55, 95% CI 1.02-2.35, p=0.040), occurrence of TE after treatment initiation (HR 2.41, 95% CI 1.76-3.29, p <0.001), Khorana score ≥2 (HR 1.25, 95% CI 1.07-1.46, p=0.004) and cancer stage (HR 1.68, 95% CI 1.25-2.26, p<0.001). The results of the univariate and multivariable Cox regression analysis are summarized in **Table 4**.

## Discussion

In this real-world cohort of patients treated with ICI, TE occurred in 16.6% of patients after a median follow up of 25 months, including 14% who developed VTE and 2.6% ATE, and were independently associated with inferior OS (HR 2.41, 95% CI 1.76–3.29; p < 0.001). These findings reinforce the emerging evidence that thrombotic events are common yet underrecognized dimensions of toxicity in the immunotherapy era (14). While early clinical trials of ICIs reported low rates of thrombotic events (approximately 2-4%), these studies were not designed to capture vascular complications and frequently excluded patients with significant comorbidity (14). Recent real word analyses have reported VTE incidence ranging from 9-24% and 2-5% ATE, aligning with the incidence observed in our cohort (15).

Venous events predominated during the early months following ICI initiation, with 41 events occurring in the first 6 months, representing 39% of all VTEs. VTEs continued to accumulate over time, increasing to 62, 73, and 79 events across subsequent 6-month intervals up to 24 months. Early VTE may reflect the interaction between malignancy associated hypercoagulability and acute immune activation triggered by treatment initiation. In contrast, arterial events were initially infrequent, with only 7 events in the first 6 months, but increased over time, reaching 14 of 20 total events (70%) in the 18–24 months period. The progressive increase of arterial events over time may reflect cumulative endothelial inflammation, accelerated atherosclerosis, or plaque destabilization driven by sustained immune stimulation. This dynamic pattern supports the hypothesis that vascular toxicity in the setting of ICI therapy may evolve over time (10, 16).

The identification of prior TE, lower baseline hemoglobin, and older age at treatment initiation as independent predictors of TE is consistent with established thrombotic risk factors but may also intersect with immune-mediated mechanisms, providing a framework for risk stratification (17). A history of TE likely reflects an underlying prothrombotic predisposition that may be amplified during immune activation (15). Baseline anemia may represent systemic inflammation, higher tumor burden, or diminished physiologic reserve, each of which may contribute to endothelial dysfunction and hypercoagulability (18, 19). Age related vascular changes and immunosenescence may further predispose older patients to immune mediated endothelial injury (20, 21). The combination of these factors during checkpoint blockade may create a permissive biological environment for thrombosis.

Beyond incidence, the association between thrombotic events and inferior survival is notable. Thrombosis remained independently associated with worse OS after adjustment for age, prior TE, hemoglobin, performance status, disease stage and other clinical covariates. This suggests that thrombotic events may represent a distinct high risk phenotype, rather than a mere reflection of advanced malignancy. Prior studies in the chemotherapy era established thrombosis as a marker of aggressive tumor biology and systemic inflammation (22). However, in the context of immune checkpoint inhibitors, the relationship may be complex. The disruption of PD-1/PD-L1 and CTLA signaling pathways by checkpoint inhibitors enhance T cell activation, augmenting antitumor immunity but also amplifying systemic inflammatory responses (23). Activated T cells release proinflammatory cytokines, including interferon-γ and tumor necrosis factor-α, which promote endothelial activation, upregulation of adhesion molecules, and increased tissue factor expression (24, 25). These processes contribute to a procoagulant state characterized by endothelial dysfunction, platelet activation, and potential amplification of neutrophil extracellular trap formation, central components of immuno-thrombosis (23, 26).

Similarly, in this cohort, patients with a Khorana score ≥2 experienced significantly worse survival (HR 1.25, 95% CI 1.07-1.46, p=0.004), yet the score did not predict TE occurrence (**Table 3**), indicting that its OS association may reflect general prognostic rather than thrombosis-specific effects. This pattern is consistent with prior work suggesting that the Khorana score captures tumor burden, anemia, and systemic inflammation, functioning as a prognostic index that identifies a subgroup of patients with more aggressive disease biology or heightened systemic inflammation. This observation is consistent with prior studies in the chemotherapy era, where elevated Khorana scores have been linked not only to thrombosis but also to poorer oncologic outcomes (27, 28).

Beyond individual clinical biomarkers, the advanced stage of cancer constitutes a significant factor that considerably deteriorates survival outcomes for patients experiencing thromboembolic complications during checkpoint inhibitor therapy. In melanoma cohorts, a higher disease stage at treatment initiation markedly exacerbates the adverse prognosis associated with subsequent thrombotic events, culminating in a substantial reduction in overall survival (28). Multi-tumor registry analyses further support that an advanced stage of cancer independently leads to the worst clinical outcomes following immune checkpoint inhibitor-associated thrombosis (29).

Our study adds to the literature by providing extended follow up, characterization of both venous and arterial thromboembolism, and multivariable adjusted survival analyses in a real-world setting. To our knowledge, this represents one of the largest and longest follow up studies in the Middle East and North Africa (MENA) evaluating thrombotic events in ICI treated patients, providing region specific insights while confirming trends observed globally. In the MENA region, high rates of smoking and metabolic risk factors may synergize with ICI-induced inflammation and prevalent inherited thrombophilia, like Factor V Leiden, to necessitate region-specific screening and further investigation (30). The retrospective design of this study limits the accuracy of incidence estimation due to potential selection bias, incomplete data capture, and lack of standardized follow-up. Nevertheless, we calculated incidence based on the available dataset to provide an approximate estimate within these inherent constraints. In addition to its retrospective single-center design, our analysis lacked inflammatory biomarkers and tumor-specific molecular characteristics, which were not uniformly available. Additionally, heterogeneity across tumor types and ICI regimens may contribute to variability in thrombotic risk.

These findings have several important clinical implications. They highlight the need for increased awareness and systematic surveillance for thrombotic complications in patients receiving immune checkpoint inhibitors, particularly among patients with prior thrombotic events, anemia, or advanced age, with special emphasis on the first two years of treatment, as the majority of events occur within 24 months. However, the optimal approach to thromboprophylaxis in patients receiving immunotherapy remains undefined, and the balance between thrombotic and bleeding risk requires careful consideration. Future research integrating inflammatory biomarkers, and endothelial activation markers may help clarify the biological mechanisms linking immune checkpoint blockade and thrombosis. Such approaches may allow more precise risk stratification and personalized thromboprophylaxis strategies in the immunotherapy era.

## Conclusions

In summary, thrombotic events are common in patients receiving immune checkpoint inhibitors and are independently associated with inferior survival. The temporal evolution from early venous to later arterial events support a model of dynamic immune-mediated vascular injury superimposed on baseline cancer-associated hypercoagulability. These findings underscore the intersection between immunotherapy and vascular biology and highlight the need for prospective mechanistic studies and risk-adapted management strategies in the emerging field of cardio-immuno-oncology.

## Data Availability

The data analyzed in this study is subject to the following licenses/restrictions: The data contain sensitive clinical information derived from electronic medical records, and access is therefore restricted. All patients data were de-identified. Data may be made available from the corresponding author upon reasonable request. Institutional Review Board (IRB) approval was obtained in accordance with ethical and privacy guidelines. Requests to access these datasets should be directed to Firas Kreidieh, fk30@aub.edu.lb.
