# Supplementary material for: Thrombotic complications in patients treated with immune checkpoint inhibitors: a retrospective study of incidence, risk factors, and survival outcomes
Source: Front Immunol. 2026 Aug 14;17:1852772. doi: 10.3389/fimmu.2026.1852772 (PMC13521917; doi:10.3389/fimmu.2026.1852772)
Supplement: Supplementary file 1 [file Table1.docx]

| **Variable** | **Univariate** | |
| --- | --- | --- |
|  | **HR (95% CI)** | **p-value** |
| Gender | 1.03 (0.72-1.47) | 0.85 |
| BMI | 1.01 (0.97-1.04) | 0.59 |
| Smoking History | 0.94 (0.63-1.39) | 0.94 |
| Age at initiating immunotherapy | 1.02 (1.01-1.04) | <0.001 |
| Surgery for primary tumor | 0.79 (0.55-1.14) | 0.22 |
| Concurrent Targeted  Therapy | 0.97 (0.54-1.72) | 0.92 |
| History of any prior TE event | 2.15 (1.33-3.48) | 0.002 |
| History of Dyslipidemia | 0.93 (0.64-1.35) | 0.72 |
| History of Diabetes Mellitus type II | 1.02 (0.70-1.49) | 0.90 |
| History of hypertension | 1.32 (0.93-1.87) | 0.12 |
| History of chronic kidney disease | 1.35 (0.87-2.00) | 0.17 |
| History of atrial fibrillation | 2.02 (1.24-3.31) | 0.005 |
| History of chronic artery disease | 0.82 (0.51-1.32) | 0.42 |
| Khorana score≥2 | 1.14 (0.94-1.37) | 0.16 |
| Anticoagulation use | 1.18 (0.99-1.39) | 0.51 |
| Antiplatelet use | 0.77 (0.50-1.18) | 0.23 |
| Hemoglobin level at treatment initiation | 0.86 (0.78-0.95) | 0.005 |
| Platelet level at treatment initiation | 1.00 (1.00-1.00) | 0.87 |

Supplementary Table 1. Univariate Cox Regression Analysis of Factors Associated with Thromboembolic Event Risk in Patients Receiving Immune Checkpoint Inhibitors
