# Supplementary material for: Thrombotic complications in patients treated with immune checkpoint inhibitors: a retrospective study of incidence, risk factors, and survival outcomes
Source: Front Immunol. 2026 Aug 14;17:1852772. doi: 10.3389/fimmu.2026.1852772 (PMC13521917; doi:10.3389/fimmu.2026.1852772)
Supplement: Supplementary file 2 [file Table2.docx]

Supplementary table: *Univariate Cox Regression Analysis of all Factors Associated with Overall Survival*

| **Variable** | **Univariate** | |
| --- | --- | --- |
|  | **HR (95% CI)** | **p-value** |
| Gender | 0.92 (0.70-1.21) | 0.56 |
| BMI | 0.97 (0.95-1.00) | 0.14 |
| Smoking History | 0.93 (0.69-1.26) | 0.67 |
| Age at initiating immunotherapy | 1.01 (0.99-1.02) | 0.08 |
| History of Dyslipidemia | 1.22 (0.93-1.61) | 0.14 |
| History of Diabetes Mellitus type II | 1.06 (0.81-1.42) | 0.65 |
| History of chronic kidney disease | 1.27 (0.90-1.80) | 0.162 |
| History of coronary artery disease | 1.16 (0.84-1.60) | 0.35 |
| Hemoglobin level at treatment initiation | 0.83 (0.77-0.89) | <0.001 |
| Platelet level at treatment initiation | 1.00 (1.00-1.00) | 0.43 |
| History of any prior TE event | 2.23 (1.55-3.19) | <0.001 |
| Occurrence of TE after treatment initiation | 2.69 (2.02-3.59) | <0.001 |
| History of hypertension | 1.36 (1.03-1.78) | 0.02 |
| History of atrial fibrillation | 1.682 (1.12-2.50) | 0.01 |
| Khorana score≥2 | 1.70 (1.11-2.67) | 0.014 |
| Diagnosis | 1.01 (0.99-1.02) | 0.23 |
| Cancer Stage | 1.84 (1.38-2.45) | <0.001 |
