# Supplementary material for: Thrombotic complications in patients treated with immune checkpoint inhibitors: a retrospective study of incidence, risk factors, and survival outcomes
Source: Front Immunol. 2026 Aug 14;17:1852772. doi: 10.3389/fimmu.2026.1852772 (PMC13521917; doi:10.3389/fimmu.2026.1852772)
Supplement: Supplementary file 3 [file Table3.docx]

Supplementary table 3: Numbers at risk at different time intervals.

| **Time point** | **No-TE at risk** | **TE at risk** | **KM S(t) no-TE** |
| --- | --- | --- | --- |
| 12 months | 395 | — | 0.850 |
| 24 months | 235 | — | 0.752 |
| 36 months | 145 | — | 0.710 |
| 48 months | 94 | — | 0.651 |
| **60 months** | **73** | **6** | **0.635** |
| **72 months** | **36** | **5** | **0.588** |
| **84 months** | **13** | **1** | **0.440** |
